# Kinematic Measures during a Clinical Diagnostic Technique for Human Neck Disorder: Inter- and Intraexaminer Comparisons

**DOI:** 10.1155/2013/950719

**Published:** 2013-02-16

**Authors:** Joseph Vorro, Tamara R. Bush, Brad Rutledge, Mingfei Li

**Affiliations:** ^1^Department of Family Medicine, College of Osteopathic Medicine, Michigan State University, East Lansing, MI 48824, USA; ^2^Department of Mechanical Engineering, College of Engineering, Michigan State University, East Lansing, MI 48824, USA; ^3^Biomechanics Division, MEA Forensic Engineers & Scientists, Laguna Hills, CA 92653, USA; ^4^Department of Mathematical Sciences and Center for Quantitative Analysis, Bentley University, Waltham, MA 02452, USA

## Abstract

Diagnoses of human musculoskeletal dysfunction of the cervical spine are indicated by palpable clues of a patient's structural compliance/noncompliance as this body segment responds to diagnostic motion demands applied by a clinician. This process includes assessments of motion range, motion performance, and changes in tissue responses. However, biomechanical quantification of these diagnostic actions and their reproducible components is lacking. As a result, this study sought to use objective kinematic measures to capture aspects of the diagnostic process to compare inter- and intraexaminer motion behaviors when performing a specific clinical diagnostic protocol. Pain-free volunteers and a group determined to be symptomatic based on a psychometric pain score were examined by two clinicians while three-dimensional kinematic data were collected. Intraexaminer diagnostic motion ranges of cervical lateral flexion and secondary rotations were consistent for each examiner and for each subject group. However, interexaminer comparisons for motion range, secondary rotations, and average velocities yielded consistently larger measures for one examiner for both subject groups (*P* < 0.05). This research demonstrates that fundamental aspects of the clinical diagnostic process for human neck disorders can be identified and measured using kinematic parameters. Further, these objective data have the potential to be linked to clinical decision making.

## 1. Introduction

Three-dimensional (3D) kinematic measures are commonly used in the diagnostic assessment of human gait [1]. These measures are collected using specialized motion capture systems with retroreflective targets on key anatomical landmarks. Data related to the locations of these targets in 3D space are used to compute joint angles, velocity measures, and other objective measures. Results from this type of research have also successfully related these kinematic parameters to general function, aging, disease, and dysfunction. However, similar use of this technology for other body regions to identify and relate dynamic 3D kinematic assessments to clinical diagnoses has been limited.

One area that has the potential to benefit from kinematic-based biomechanical parameters is the study of musculoskeletal disorders (MSDs) of the cervical spine. MSDs of the cervical spine have a significant impact on society, frequently causing major disabilities and lasting functional limitations [2–4]. These disorders affect as many as two-thirds of the world's population and are second only to low back problems [5, 6]. A unique aspect associated with these neck disorders is that they are commonly diagnosed by palpation, which are clinician-directed techniques (passively induced motions) guided by physical clues produced by patient's regional physiologic responses [7]. Specifically, during these procedures, a clinician monitors a patient using light, nonintrusive touch, while continually assessing compliance/noncompliance of the anatomical structures as they respond to the motion demands of the clinical technique. The results can be deviations from normative ranges of motion (ROM) and changes in tissue resistances, particularly at the end of motion range. 

However, many of these structural diagnostic techniques lack objective, scientific, evidence for their relevance and effectiveness. Attempts to develop reliable approaches to these clinical evaluations exist; however, none have yet captured essential components of the diagnostic procedures [8, 9]. As a result, a need exists to quantify clinician-directed diagnostic actions into components that are measurable and also meaningful to the diagnostic process, clinical decision making, and evaluation of treatment effectiveness. 

Therefore, the purpose of this study was to use 3D kinematic measures to quantify and compare inter- and intraexaminer motion behaviors when performing a cervical diagnostic protocol on a group of symptom-free volunteers, and a group determined to be symptomatic based on a psychometric pain score. Specifically, three kinematic parameters were used to evaluate the diagnostic process: (1) the magnitude and variation of the primary diagnostic motion (lateral cervical flexion), (2) the magnitude and variation of secondary rotations that occurred at maximal diagnostic ROMs, and (3) the angular velocities (speeds) with which the examiners performed the passive diagnostic tests.

## 2. Methods

### 2.1. Subjects and Subject Group Assignment

Subjects were recruited from a university campus clinical center, and from the general university student, faculty, and staff populations. These volunteers provided written consent prior to participation. Everyone completed two questionnaires: a Visual-Analog Scale (VAS) [10] to determine the level of pain in the neck region and a Neck Pain and Disability Scale (NPDS) [11] to quantify the level of dysfunction. 

As part of the protocol to establish subject groups, all volunteers also received an initial (reference) diagnostic assessment by a physician (Examiner 1) using the standard palpatory diagnostic test of right and left cervical lateral flexions (side-bending). Examiner 1 was blinded to the VAS and NPDS scores. 

Based on this assessment by Examiner 1, and the results of the VAS questionnaire, two subject groups were established.

(1) *Control Group*: subjects who self-scored the VAS = 0, and who were symmetric for the right and left lateral flexion diagnostic motions as determined by Examiner 1.

(2) *Experimental Group*: subjects who self-scored the VAS ≥ 3 indicating cervical pain [10].

Evaluations were conducted on 131 total volunteers. Of these, 41 qualified for the study, including control group (*n* = 22; 16 males, 4 females and 2 who opted not to provide a response) and experimental group (*n* = 19; 14 males and 5 females). Volunteers not qualifying for the study were dismissed.

### 2.2. Additional Diagnostic Evaluations by Examiners 2 and 3

To be able to evaluate inter- and intraexaminer kinematic consistencies during the specific diagnostic test, control and experimental subjects experienced further diagnostic testing (cervical lateral flexion) from two additional physicians (Examiners 2 and 3). Both Examiners conducted the passive diagnostic motions in front of a six-camera motion capture system (Qualisys, Gothenburg, Sweden) while head and neck motions relative to the thorax were recorded (detailed in Section 2.3).

Examiners 2 and 3 were blinded to each subject's VAS and NPDS scores, subject diagnostic categories, and each other's assessments. Two separate diagnostic trials (Trial 1 and Trial 2) were conducted by each examiner, with each trial consisting of three right and left repeated motions. 

**Figure 1:**
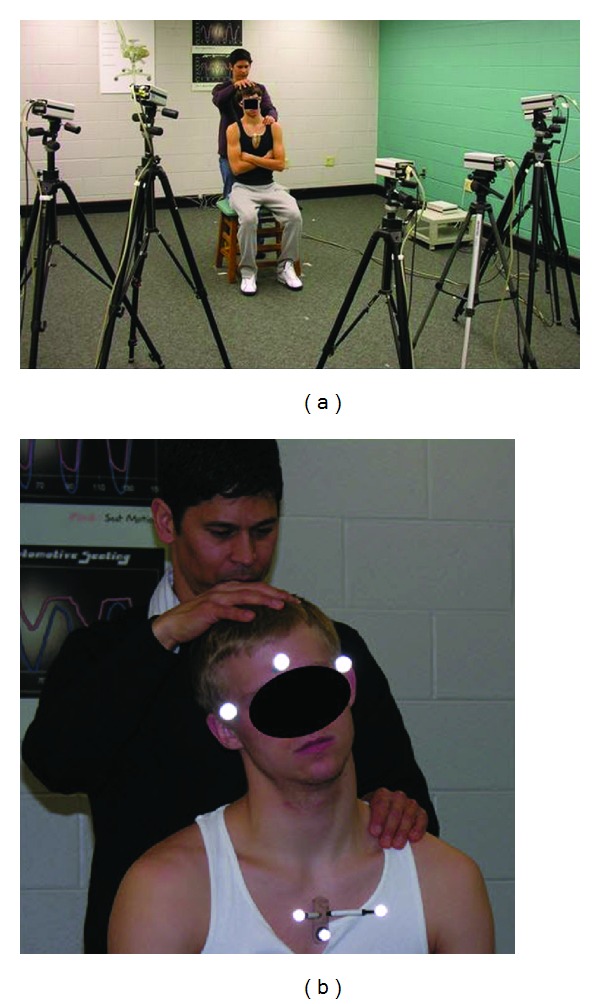
(a) Examiner and subject positions, with motion capture cameras in place. (b) Retroreflective marker positions during execution of the lateral flexion diagnostic test.

The cervical palpatory diagnostic technique used by all three examiners during all subject screening and testing is a commonly practiced, standard test of cervical lateral flexion [12, 13]. Each examiner was a practicing osteopathic physician specialized in manual medicine for over 10 years. The procedure was as follows:

1. the examiner aligned himself/herself posterior to a seated subject. Each subject had to remain passive as the diagnostic motions were performed (Figures 1(a) and 1(b)).
2. One of the examiner's hands (the moving hand) was placed gently on a subject's head, while the contralateral hand was placed lightly on the posterior thoracic midline.
3. The examiner passively guided the subject's head in lateral flexion to the right, taking the right ear toward the ipsilateral shoulder until a palpable sense of end ROM was achieved. End ROM was defined as the point where a tissue texture change required a substantial increase in force to continue the diagnostic motion [13].
4. At the conclusion of the initial motion, the subject's head was guided back to neutral, the examiner's hand placement was changed, and movement to the contralateral side was conducted.
5. The subject's head was guided back to neutral again, and steps 2–5 were repeated so that one trial consisted of three right lateral flexions and three left lateral flexions.

Examiners then made their clinical evaluations based upon the following, previously established criteria [10, 12, 13]:

1. visual and proprioceptive evaluations of the magnitude and symmetry of right and left cervical lateral flexions,
2. palpatory assessments from the moving hand to determine quality of motion, specifically, smoothness and tissue resistance, and
3. “end-feel” was considered as any specific resistance to the diagnostic movements.

### 2.3. Kinematic Data Collection

A six-camera Qualisys motion capture system, in conjunction with retroreflective markers, was used to capture motions of the head relative to the thorax (Figure 1(a)). Retroreflective markers were placed on each subject's temples, and one marker was centered on the forehead to capture head motions. Three additional markers, in the form of a rigid triad, were attached to the skin over the central sternum of each subject (Figure 1(b)). The duration of each trial was not controlled, so that examiners moved the subjects at their preferred motion rates for the diagnostic process. Test trial times ranged from 30 to 65 seconds, and data were collected at 20 Hz.

To establish subject-specific neutral head reference locations (0° angle), each trial began with a three-second period where baseline data were collected as subjects were instructed to remain still and face forward. After baseline data were collected, the examiner's hands were placed on the subject to begin the diagnostic movements.

**Figure 2:**
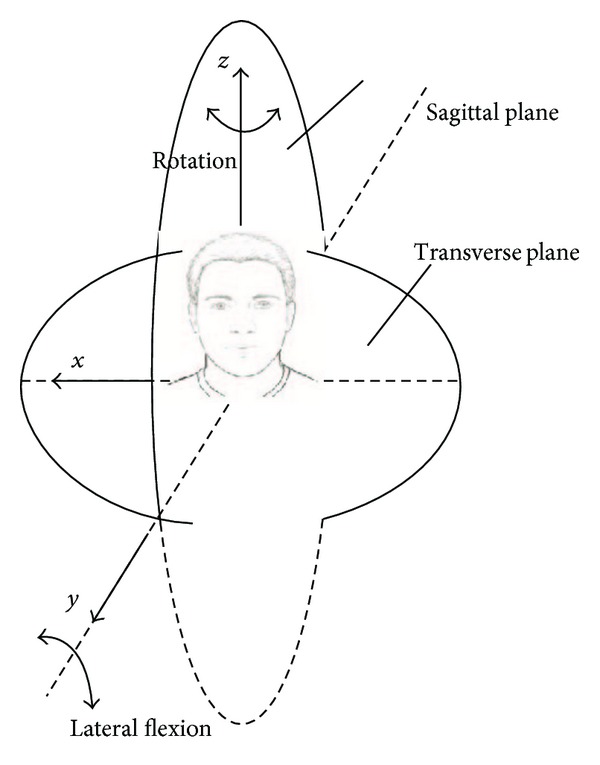
Lateral flexion and coupled axial rotations of the head during the diagnostic test.

The motion capture system was calibrated daily with the global reference system oriented such that the *x*-axis progressed horizontally from the equivalent of a subject's left to right, the *y*-axis from posterior to anterior, and the *z*-axis vertically from inferior to superior (Figure 2). The system error was less than ±2 mm and ±1°.

### 2.4. Data Analysis

Euler angles were used to compute motions of the head relative to the thorax [14]. Thus, any movement of the thorax was accounted for by the relative segment assessment. First, local Cartesian coordinate systems were established on the head and thorax in the form of unit vectors ($\hat{i}$, $\hat{j}$, and $\hat{k}$). These coordinate systems were generated from the coordinates of the markers on the head (forehead, left temple, and right temple) and the sternum (middle sternum, left sternum, and right sternum). These local coordinate systems were then aligned with respect to the global coordinate system. Based on the unit vectors for a local coordinate system at frame ‘‘*n*” and frame ‘‘*n* + 1,” the rotation matrix between the two frames could be calculated, and, thus, the angles of rotation could be determined. The rotation matrix, based upon a rotation sequence of *yzx*, was determined from the summation of the rotation matrices for rotations around the *y*-axis, *z*-axis, and *x*-axis independently as follows:

(1) $$\begin{array}{c} R_{y}\left(\theta_{1}\right)=\left[\begin{array}{ccc} \cos\theta_{1} & 0 & \sin\theta_{1}\\ 0 & 1 & 0\\ -\sin\theta_{1} & 0 & \cos\theta_{1} \end{array}\right],\\ R_{z}\left(\theta_{2}\right)=\left[\begin{array}{ccc} \cos\theta_{2} & -\sin\theta_{2} & 0\\ \sin\theta_{2} & \cos\theta_{2} & 0\\ 0 & 0 & 1 \end{array}\right],\\ R_{x}\left(\theta_{3}\right)=\left[\begin{array}{ccc} 1 & 0 & 0\\ 0 & \cos\theta_{3} & -\sin\theta_{3}\\ 0 & \sin\theta_{3} & \cos\theta_{3} \end{array}\right],\\ R\left(\theta_{1},\theta_{2},\theta_{3}\right)=R_{y}\left(\theta_{1}\right)R_{z}\left(\theta_{2}\right)R_{x}\left(\theta_{3}\right)=\left[R\right], \end{array}$$

where *θ*_1_ was the angle of rotation about the *y*-axis (lateral flexion), *θ*_2_ was the angle of rotation around the *z*-axis (axial rotation), and *θ*_3_, was the angle of rotation around the *x*-axis (flexion and extension) [15]. The rotation matrix, *R*(*θ*_1_, *θ*_2_, *θ*_3_), was then multiplied with the unit vectors of the local coordinate system at the original frame, *X*_*n*_, to determine the location of the local coordinate system in the next frame, *X*_*n*+1_, allowing the computation of the three angles throughout the entire diagnostic motion. Although three angles were computed, forward flexion and extension data were not analyzed since the focus of the palpatory diagnostic movement was on lateral flexion and rotation (also the largest two motions) (Figure 2). Three variables were analyzed.

#### 2.4.1. Cervical ROM

Maximal diagnostic right and left motions were identified as angles greater than 10° that were greater than the previous 10 values and greater than the following 10 values. Additionally, total diagnostic ROMs (from maximum right to maximum left) were computed. All angles were based on the subject's self-selected neutral position (0° angle).

#### 2.4.2. Secondary Rotations

Axial rotations at frames corresponding to maximum right and left diagnostic ROMs were identified for each subject and averaged for each examiner. Additionally, the total rotation (from maximum diagnostic right to maximum diagnostic left) was computed. Positive axial rotations were associated with ipsilateral lateral flexions such that right lateral flexion ROM was associated with axial rotation to the right, and left lateral flexion ROM was accompanied by axial rotation to the left. Negative axial rotations indicated contralateral rotation, or axial rotation in the opposite direction to the lateral flexion being performed (Figure 2). 

#### 2.4.3. Diagnostic Motion Angular Velocities

Angular velocities (degrees/second) for each passive diagnostic motion were identified as the slopes of linear regressions calculated from the start-to-peak excursions for each motion. This portion of the curve corresponded to the movements where the examiners conducted their passive diagnoses. Average angular velocities for diagnostic movements to the right and to the left were analyzed. Additionally, the total average angular velocities (for both right and left movements) were computed and analyzed.

### 2.5. Statistical Analyses

One-way repeated measures ANOVAs assessed intraexaminer data, while *t*-tests compared interexaminer data for three kinematic factors: (1) lateral flexion ROMs, (2) secondary rotations at maximum ROM, and (3) the rates (velocities) at which the diagnostic motions were conducted.

Data for trials one and two were evaluated separately for intraexaminer comparisons so that the diagnostic motion consistencies could be assessed. However, for between-examiner comparisons, the two trials for each examiner were averaged. The criterion alpha level was set to 0.05.

## 3. Results 

### 3.1. Subjects

The average age of the control subjects was 19.9 years (±1.9) and experimental subjects 27.5 years (±13.1). A statistical analysis indicated no significant between-group age differences. The VAS for control subjects was zero (the requirement for inclusion in this subject group); experimental subjects produced an average VAS of 4.6 out of 10.0. The average NPDS score for control subjects was 2.5 (±4.1) and 46.9 (±21.0) for experimental subjects, with larger numbers indicating higher levels of head and neck disability.

### 3.2. Intraexaminer ROMs

When comparing the average total diagnostic ROM, Examiner 2 demonstrated between-trial differences of less than 1° for the control group and less than 3° for the experimental group; while Examiner 3's passive, diagnostic ROMs varied less than 1° for both subject groups (Tables 1 and 2). One-way ANOVAs indicated no within-examiner ROM differences for right, left, or total diagnostic ROM or subject group, and no differences for the repeated trials.

### 3.3. Intraexaminer Secondary Rotations

The total secondary rotations produced by each Examiner also demonstrated slight differences across repeat trials. Examiner 2 produced less than 1° of secondary rotation between trials for the control group and less than 3° for the experimental group. Examiner 3's passive between-trial secondary rotations were less than 1° for control subjects and slightly more than 1° for the experimental subjects (Tables 3 and 4). One-way ANOVAs indicated no within-examiner secondary rotations differences.

### 3.4. Intraexaminer Rates of Motion

**Figure 3:**
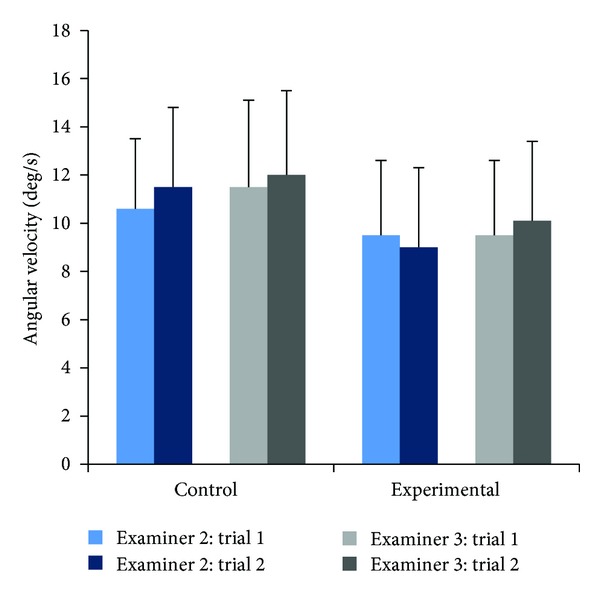
Angular velocities for diagnostic tests, subject group, trials and examiners. Data represent average velocities for each trial for all subjects. Bars represent one standard deviation.

Assessments of within-examiner diagnostic angular velocities indicated that on average both examiners produced between-trial differences of less than 1.0 degree/sec for both control and experimental subjects (Tables 5 and 6, Figure 3).

One-way repeated ANOVAs indicated differences in the velocity data; however, they were not consistent across examiner, trial, or group. Specifically, Examiner 2 produced significantly different between-trial velocities (*P* = 0.045) when moving control subjects to the right. Further, Examiner 2 produced significantly different between-trial velocities when moving experimental subjects to the right (*P* = 0.030 and to the left (*P* = 0.044). Examiner 3 produced significantly different between-trail velocities when moving control subjects only, to the right and left (*P* = 0.006 and *P* = 0.035, resp.).

### 3.5. Interexaminer ROM

**Figure 4:**
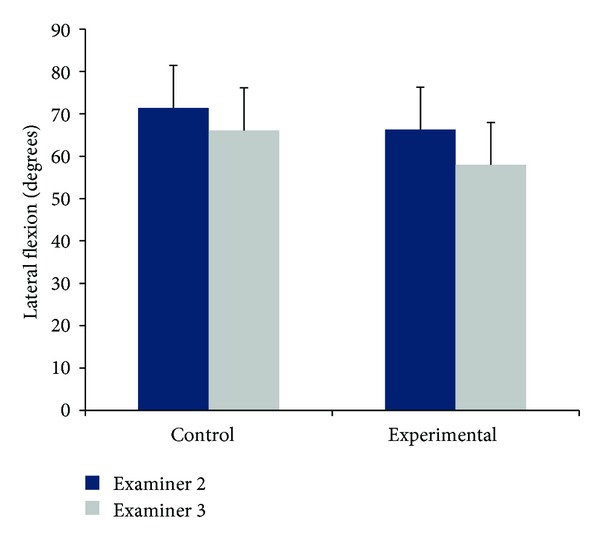
Comparisons between Examiner 2 (dark blue) and Examiner 3 (grey) for average total passive diagnostic ROM. Bars represent one standard deviation.

Between-examiner comparisons for diagnostic ROM indicated that Examiner 2 consistently moved control and experimental subjects through greater ranges than Examiner 3 (average percent difference between examiners: for the control group = 7.4%; for the experimental group = 12.4%) (Tables 1 and 2, Figure 4). Additionally, both examiners produced greater total diagnostic ROM for control subjects.

Independent samples *t*-tests indicated significant differences in between-Examiner passive motions for experimental subjects only, right ROM (*P* = 0.029), left ROM (*P* = 0.053), and total ROM (*P* = 0.040).

### 3.6. Interexaminer Secondary Rotations

**Figure 5:**
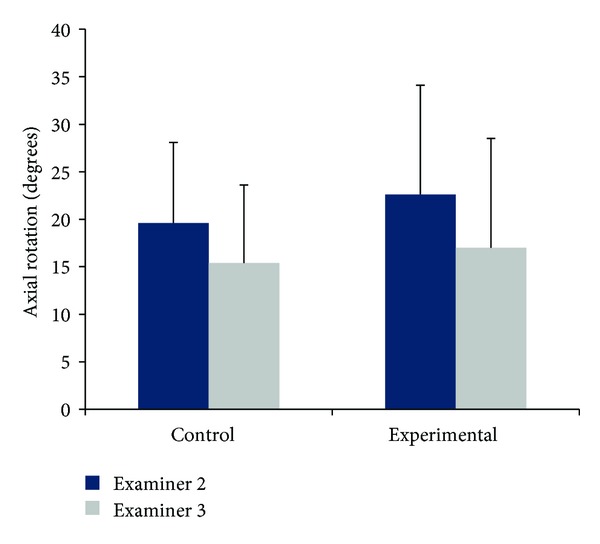
Comparisons between Examiner 2 (dark blue) and Examiner 3 (grey) for average secondary rotations. Bars represent one standard deviation.

Secondary rotations for all trials, Examiners and subject groups are presented in Tables 3 and 4. Between-examiner comparisons indicated that Examiner 2 consistently produced greater average secondary rotations than Examiner 3 (Tables 3 and 4, Figure 5). The average percent difference between examiners for the control group was 21.3% and 25.1% for the experimental group. In addition, both Examiners produced greater secondary rotations when passively moving experimental subjects.

Independent samples *t*-tests indicated significant between-examiner differences for passive secondary rotations for control subjects only: for diagnostic motions to the right (*P* = 0.036), to the left (*P* = 0.026), and for total right and left motions (*P* = 0.018). No between-examiner differences for secondary rotations occurred for experimental subjects.

### 3.7. Interexaminer Rates of Motion

The right, left, and total average velocities for control and experimental subjects for each examiner are presented in (Tables 5 and 6). Examiner 3 consistently produced higher average diagnostic motion velocities than Examiner 2. Greater angular velocities were produced by each examiner during diagnostic motions for control subjects. 

Between-examiner average angular velocities varied less than 6% for diagnostic motions and subject groups. Independent samples *t*-tests indicated a significant difference between Examiners for average velocities to the left for Control subjects (*P* = 0.050).

## 4. Discussion

Manual diagnostic assessments of human neck motions are a standard part of the clinical examination for patients suffering from neck disorders, neck pain, and discomfort. However, little biomechanical evidence exists that quantifies these clinician-directed palpatory diagnostic techniques and comparisons across subject groups and different examiners. 

As a result, this study used kinematic measures to capture biomechanical aspects of a standard clinical screening test (cervical lateral flexion) so that inter- and intraexaminer diagnostic motions could be compared through objective data sets. Control subjects (*n* = 22) demonstrated diagnostic motion symmetry as determined by a trained, experienced examiner. These subjects also self-reported no pain based on results from a psychometric test. Experimental subjects (*n* = 19) consisted of volunteers with positive scores of ≥3 from the same psychometric test indicating neck pain.

Two additional examiners (2 and 3) conducted passive diagnostic motions for each subject in front of a motion capture system. Three specific parameters were evaluated from the kinematic data: diagnostic ROM, secondary rotations around the primary diagnostic motion, and angular velocities.

Intraexaminer data for two kinematic variables, total diagnostic ROM, and secondary rotations were consistent within each examiner's data sets and yielded no significant differences. The statistical analysis did indicate several differences in velocity data; however, they were not consistent across examiner, trial, or subject group. 

Interexaminer kinematic data varied. Examiner 2 consistently produced greater average passive diagnostic ROMs than Examiner 3, and Examiner 2 produced greater average secondary rotations. Between-examiner velocities were not significantly different except for one comparison, movement to the left for control subjects.

Modern medicine uses multiple standard, objective, evidence-based tests to determine and confirm the presence of disease, for example, microbiological cultures, blood samples and medical images. These tests are used to the extent needed to establish an accurate medical diagnosis. Once the diagnosis is made, appropriate treatments can be implemented, and prevention options can be sought.

While laboratory tests and clinical evaluations make up the bulk of medical diagnostic techniques for most of the body's organ systems, the health and integrity of the musculoskeletal system is typically evaluated via palpation. This structural (haptic) diagnosis uses passive gross motions introduced as tests to gain palpable information about the body's regional and segmental motor functions and integrity. 

Review articles indicate that much of the related literature is associated with manual medicine treatment techniques, and the majority is qualitative in nature. However, while some excellent manual medicine diagnostic studies exist, review articles indicate that findings often vary between studies because of methodological differences, different clinician experiences, lack of examiner training and consensus before the study, lack of concomitant comparisons between symptomatic and asymptomatic subjects, and lack of objective markers [7, 16–19]. Further, many studies of inter- and intraexaminer comparisons do not use clinically relevant diagnostic tests (i.e., the guiding of a body part to a palpable (comfortable) sense of diagnostic motion end range) [13], but rather required the production of maximal anatomical joint excursions [20–23].

No within-examiner ROM differences for movements, subject group, or repeated trials were observed in this experiment. Within-examiner consistencies of this nature are well documented in the literature [7, 24–26]. However, a few within-examiner differences occurred for average angular velocities. Also, when evaluating the subject groups, Examiners consistently moved the experimental group slower than the control group. It is suggested that the reason for the observed average velocity differences in this paper relates specifically to the clinical intent of the palpatory process. The two examiners performed the diagnostic motions while simultaneously monitoring the motions for specific palpable cues, for example, tissue texture changes and restrictive barriers. As a result, the examiners moved the pain group at a slower rate, even though they were blinded to the group assignment and they did not have any verbal communication with the subjects. Other studies have looked at angular velocities; however, they differed from this paper in that the motions were active instead of passive, and, motions other than cervical lateral flexion were evaluated. For example, Sjölander et al. [27] and Prushansky et al. [28] studied active cervical motions for a group of acute whiplash patients and no-pain controls and found reduced peak velocities for the whiplash groups. Bahat et al. [29] used a virtual reality motion assessment system to evaluate pain and no-pain subjects and reported reductions in both peak and average velocities for motions produced by pain subjects. However, this group only studied cervical sagittal flexion/extension and rotation. 

The current findings also indicated that between-examiner diagnostic ROM, secondary rotations and average motion velocities varied. Interexaminer differences of this nature are well reported [7, 16–19, 30]. However, these papers point out that the comparisons were typically made on qualitative assessments, and that reasons for the reported differences were due to the lack of objective markers by which to make evaluations.

A few studies have made objective measurements of clinical cervical motions with between-examiner comparisons. Lantz et al. [31] reported good between-examiner agreement for cervical axial rotations and lateral flexions; however, for normal subjects only, for maximal ROMs instead of diagnostic ROMs, and their protocol required the use of a thoracic harness during testing which is not a standard clinical protocol. Morphett et al. [32] also reported good between-examiner ROM agreement for both pain and nonpain subjects, although their protocol required maximal cervical motion ranges. Strimpakos et al. [26] examined maximal ROM for nonpain subjects only and found that one examiner yielded significantly lower neck ROM values in all assessments than the other examiner. Further, their subjects were fitted to a stabilization system that isolated the cervical region from the rest of the body, and there were no reports of secondary motions or associated velocities.

In terms of general kinematic differences between pain and no-pain subjects, two of the three variables in this experiment, diagnostic ROM and average motion velocities were reduced for the pain subjects as compared to the nonpain group and supported previous research [21, 29, 33–37]. The third kinematic variable, secondary rotations, were greater for pain subjects, directly supporting a previous investigation [33], while also supporting additional work that included different experimental intentions and protocols [20, 25, 26, 34, 38–41].

The results presented here indicate that diagnostic techniques for MSD have measurable anatomical and physiological characteristics. As a result, MSD yields objective measures that manifest as altered motion qualities detected through palpation, for example, differences in motion range and motion symmetry, modified proprioception, changes in muscle recruitment patterns, and altered kinematics.

The importance and necessity of this work are confirmed by numerous review articles that document contradicting results, while also indicating the need for objective measures to eliminate inconsistencies found from subjective measures [7, 16–19]. In addition, the future potential of this work can be seen in training new clinicians—specifically, using objective information to help learners understand diagnostic movements, developing consistent approaches to conducting these assessments, and relating specific changes in kinematic measures to levels of dysfunction to assist with the diagnosis, and to document treatment effects.

## Figures and Tables

**Table 1:** Examiner 2, comparisons of diagnostic motions for control and experimental subjects for right, left, and total passive (maximum right to left) diagnostic ROM.

Subjects	Right diagnostic ROM		Left diagnostic ROM		Total diagnostic ROM	
	Trial 1	Trial 2	Trial 1	Trial 2	Trial 1	Trial 2
	Ave (SD)		Ave (SD)		Ave (SD)	
	(Degrees)		(Degrees)		(Degrees)	
Control	35.6	36.2	35.5	35.5	71.1	71.7
	(7.2)	(6.7)	(5.6)	(5.7)	(11.6)	(11.5)
Experimental	33.0	31.8	34.6	33.0	67.6	64.8
	(7.1)	(6.2)	(7.0)	(6.3)	(12.2)	(11.8)

**Table 2:** Examiner 3, comparisons of diagnostic motions for control and experimental subjects for right, left, and total passive (maximum right to left) diagnostic ROM.

Subjects	Right Diagnostic ROM		Left Diagnostic ROM		Total Diagnostic ROM	
	Trial 1	Trial 2	Trial 1	Trial 2	Trial 1	Trial 2
	Ave (SD)		Ave (SD)		Ave (SD)	
	(Degrees)		(Degrees)		(Degrees)	
Control	32.6	32.7	33.1	33.6	65.7	66.3
	(7.1)	(7.1)	(6.3)	(5.9)	(11.7)	(11.3)
Experimental	28.5	28.3	29.7	29.4	58.2	57.7
	(5.8)	(6.9)	(6.7)	(6.5)	(11.4)	(12.0)

**Table 3:** Examiner 2, secondary rotations at maximum diagnostic right lateral flexion ROM, specifically, rotations at maximum right lateral flexion, at maximum left lateral flexion, and the total rotation (from maximum diagnostic right to maximum diagnostic left).

Subjects	To the right		To the left		Total rotation	
	Trial 1	Trial 2	Trial 1	Trial 2	Trial 1	Trial 2
	Ave (SD)		Ave (SD)		Ave (SD)	
	(Degrees)		(Degrees)		(Degrees)	
Control	8.8	9.3	10.8	10.4	19.7	19.6
	(4.8)	(4.3)	(4.8)	(6.1)	(8.0)	(8.8)
Experimental	10.9	12.1	10.7	11.6	21.6	23.7
	(4.5)	(5.1)	(8.4)	(8.4)	(10.9)	(11.7)

**Table 4:** Examiner 3, secondary rotations at maximum diagnostic right lateral flexion ROM, specifically, rotations at maximum right lateral flexion, at maximum left lateral flexion, and the total rotation (from maximum diagnostic right to maximum diagnostic left).

Subjects	To the right		To the left		Total rotation	
	Trial 1	Trial 2	Trial 1	Trial 2	Trial 1	Trial 2
	Ave (SD)		Ave (SD)		Ave (SD)	
	(Degrees)		(Degrees)		(Degrees)	
Control	8.7	8.5	6.7	7.0	15.4	15.5
	(4.6)	(4.8)	(5.2)	(5.2)	(7.7)	(8.5)
Experimental	11.2	9.2	6.3	7.2	17.5	16.4
	(4.6)	(4.5)	(8.0)	(8.6)	(11.1)	(11.6)

**Table 5:** Comparisons of Examiner 2 angular velocities for subject group and trials, including average angular velocities for movements to the right, left, and total average angular velocities for right and left movements.

Subjects	Right velocity		Left velocity		Total average angular velocity	
	Trial 1	Trial 2	Trial 1	Trial 2	Trial 1	Trial 2
	Ave (SD)		Ave (SD)		Ave (SD)	
	(°/second)		(°/second)		(°/second)	
Control	10.5	11.8	10.7	11.3	10.6	11.5
	(2.9)	(3.4)	(2.9)	(3.3)	(2.9)	(3.3)
Experimental	9.7	9.1	9.4	9.0	9.5	9.0
	(3.1)	(3.1)	(3.0)	(3.4)	(3.0)	(3.3)

**Table 6:** Comparisons of Examiner 3 angular velocities for subject group and trials, including average angular velocities for movements to the right, left, and total average angular velocities for right and left movements.

Subjects	Right velocity		Left velocity		Total average angular velocity	
	Ex. 2	Ex. 3	Ex. 2	Ex. 3	Ex. 2	Ex. 3
	Ave (SD)		Ave (SD)		Ave (SD)	
	(°/second)		(°/second)		(°/second)	
Control	11.1	11.6	11.8	12.3	11.5	12.0
	(3.7)	(3.2)	(3.4)	(3.5)	(3.6)	(3.4)
Experimental	9.7	10.0	9.4	10.1	9.6	10.0
	(3.2)	(3.3)	(2.9)	(3.4)	(3.6)	(3.3)
